# Mid- to Long-Term Survival of Geriatric Patients with Primary Septic Arthritis of the Shoulder: A Retrospective Study over a Period of 20 Years

**DOI:** 10.3390/jpm13071030

**Published:** 2023-06-22

**Authors:** Johannes Rüther, Lars Taubert, Kim Loose, Maximilian Willauschus, Sandeep Silawal, Michael Millrose, Hermann Josef Bail, Markus Geßlein

**Affiliations:** 1 Department of Orthopedics and Traumatology, Paracelsus Medical University, General Hospital Nuremberg, Breslauer Straße 201, 90471 Nuremberg, Germany; 2 Institute of Anatomy and Cell Biology, Paracelsus Medical University, General Hospital Nuremberg, Prof. Ernst Nathan Str. 1, 90419 Nuremberg, Germany; 3 Department of Trauma Surgery and Sports Medicine, Garmisch-Partenkirchen Medical Centre, 82467 Garmisch-Partenkirchen, Germany

**Keywords:** septic arthritis, geriatric patients, shoulder, risk factors, infection

## Abstract

Septic arthritis of the shoulder is an urgent medical emergency that often occurs in elderly patients and is associated with high morbidity and mortality. Retrospectively, 56 patients aged ≥60 years, treated for primary septic monoarthritis of the shoulder at a maximum care hospital between 1 July 2001, and 30 July 2022, were included in this study. The primary aim of the study was analyzing survival rates and different bacteria in these patients. For statistical analysis, Kaplan–Meier curves were used for survival probability and the log-rank test was used to compare a survival probability of 5 years. The mean patient age was 78.7 years and a mean follow-up time of 3011.8 days. The mean survival of the entire study population was 920.3 days or 2.5 years. Significantly impaired 5-year survival was found only with increasing age and higher American Society of Anesthesiologists (ASA) physical status (PS) classification scores. Eight different types of bacteria were detected in the synovial fluid cultures. A total of 42 of 48 overall pathogens was Gram-positive and 6 were Gram-negative bacteria. *Staphylococcus aureus* was identified as the most frequent variant. We conclude that the mean survival is significantly shortened within the first 5 years with increasing age and ASA PS classification.

## 1. Introduction

Despite current medicine, septic arthritis of the shoulder is a potentially life-threatening emergency that frequently occurs in the elderly [1,2,3]. Due to the disease’s high morbidity and mortality, a rapid diagnosis with initiation of adequate therapy is essential [2,3]. Mostly, Gram-positive bacteria enter the affected joint through surgery or hematogenous spread [2,4,5].

The incidence of septic arthritis varies significantly in different regions of the world. It varies from 2–10 cases per 100.000 population. Moreover, the incidence seems to be rising when we examine the literature. The incidence of primary septic arthritis has been rising in recent decades. Factors influencing the prevalence are an aging population, an increase in invasive joint procedures and more comorbidities of the population. A growing prevalence of antibiotic resistant bacteria leads to an even more challenging treatment of septic arthritis of the shoulder. Studies on the epidemiology of septic arthritis of the shoulder are rare, but the incidence seems to be around 4 to 6 per 100.000 per persons and year. Septic arthritis of the shoulder varies from about 3 to 5% of these cases. The incidence varies significantly in different countries. This could be due to differences in diagnostic criteria, reporting systems and reporting mechanisms. Moreover, the incidence has varied in recent decades [4,6,7,8]. For instance, in the United Kingdom the incidence increased from 5.5/100.000 in 1998 to 7.8/100.000 in 2013 with 4.3% affecting the shoulder joint. In Taiwan, the incidence increased from 9.8/100.000 in 1998 to 13.3/100.000 [9,10]. Most of the infections of primary septic arthritis affect one joint (80–90%). In 10 to 20%, more than one joint, typically two or three joints, is affected. Septic arthritis affecting many joints arises typically in multimorbid patients [6,11,12,13]. In about half of the cases, the knee joint is affected by septic arthritis [12,14,15]. The hips (15 to 25%) and the shoulders (5 to 10% of all cases) are rare locations for primary septic arthritis [14,16,17]. The ilosacral, sternoclavicular and symphysis pubica joint are locations for atypical septic arthritis [8,18,19]. Allthough there are some studies on the incidence of septic arthritis of the shoulder, there are still scarce data about primary septic arthritis of the shoulder.

The main risk factors are age, immunosuppression, previous surgery, intra-articular joint injections, a preexisting joint disease such as osteoarthritis, rheumatoid arthritis, gout, or pseudogout; diabetes mellitus; medical prosthesis, especially joint prosthesis; or skin infections [14,20,21].

Treatment of septic arthritis requires both rapid use of effective antimicrobial agents and surgical debridement of the affected joint, with priority given to offloading the joint [4,6,22,23]. Broad-spectrum antibiotics are usually administered immediately after the joint aspiration [23,24]. Arthroscopic or open-joint debridement are gold-standard treatments for septic arthritis [6,25].

Most commonly, Gram-positive skin bacteria, especially *Staphylococcus aureus* affect septic arthritis [2,7,22]. Methicillin-resistant *Staph aureus* is the causing agent in about a quarter of the cases and seems to play a key role in primary septic arthritis too [26,27]. Biofilm-forming organisms are frequently involved, as they can absent themselves from this state and cause hematogenous spread, which is frequently seen in patients with medical implants [28]. Gram-negative agents are the causing agent in 10 to 20 % of primary septic arthritis cases [16,29,30,31]. This is why every patient should undergo a microbial probe to isolate the bacteria and receive the best antibiotic treatment. Cefuroxim is the first-line treatment for patients with septic arthritis. This antibiotic has a good effect on common bacteria and can be applied intravenously to reach a high level in the blood of the patient.

The American Society of Anesthesiologists (ASA) physical status classification system is a tool with the purpose of assessing a patient’s medical comorbidities prior to anesthesia. It is classified preoperatively and provides a quick overview of the patient’s medical condition [32,33,34]. The classification system was not developed to predict perioperative risks; however, many subsequent publications have shown that ASA classification strongly correlates with outcome and has the potential ability to predict intraoperative and postoperative outcomes, such as intraoperative blood loss or postoperative infection rates [32,33,34].

The primary aim of this study was to analyze the overall survival of older adults with acute primary septic monoarthritis of the shoulder after surgical treatment. The secondary aim was to assess the causing pathogens for primary septic arthritis of the shoulder.

## 2. Materials and Methods

Patients’ data were evaluated using medical records from a specialized hospital over a period from 1 January 2001 to 31 July 2022. A total of 106 patients was admitted to the emergency department for primary septic arthritis of the shoulder. Of these, 56 patients were enrolled in the study.

The inclusion criteria for the study consisted of an age ≥60 years and a primary septic monoarthritis of the shoulder affecting the glenohumeral joint with fulfillment of at least one of the Newman criteria for septic arthritis [35].

Patients with unavailable survival data and incomplete medical records or follow-up were excluded (8 patients excluded). Patients with a periprosthetic joint infection (12 patients excluded), recent surgery or trauma (<6 months) (19 patients excluded), open skin wounds on the shoulder or general wound treatment were also excluded (1 patient excluded). Likewise, a diagnosis of rheumatoid arthritis, acute gout, or crystal arthropathies led to an exclusion (8 patients excluded). Patients with infections of joints other than the glenohumeral joint were also excluded (2 patients excluded).

Diagnostic measures included: hospital admission, clinical examination, blood examination, joint radiography, joint lavage with microbiological sampling during surgery, empiric antibiotic treatment after surgery, and targeted antibiotic therapy according to the results of microbiological examination and antibiograms.

Factors recorded included: Age in years, sex, joints involved, date of hospital admission, clinical examination methods, blood parameters (including leukocyte count, CRP, procalcitonin and blood cultures), ASA-PS classification scores at the time of surgery, microbiological blood culture analysis, time to follow-up in days and date of death.

The American Society of Anesthesiologists (ASA) physical status classification system is a tool with the purpose of assessing a patient’s medical comorbidities prior to anesthesia. It is classified preoperatively and provides a quick overview of the patient’s medical condition [32,33,34]. It is divided into six groups: ASA I: a normal healthy patient; ASA II: a patient with mild systemic disease; ASA III: a patient with severe systemic disease that is not life-threatening; ASA IV: a patient with a severe systemic disease that is a constant threat of life; ASA V: a moribund patient who is not expected to survive without an operation; ASA VI: a patient declared brain dead [32,33].

Joint aspirates were performed during the operation procedure by an orthopedic surgeon using a standard aseptic technique and then sent for examination for aerobic/anaerobic growth. Synovial fluid was inoculated onto aerobic chocolate, sheep blood agar, and anaerobic sheep blood agar plates and then incubated aerobically at 37 °C with 5% CO_2_ and anaerobically at 37 °C for 7 days. The remaining liquid was inoculated into a thioglycolate broth for sample enrichment and then incubated for 14 days. Bacteria were identified by matrix-assisted laser desorption/ionization time-of-flight mass spectrometry (MALDI-TOF MS). Antibiotic susceptibility testing was performed according to the European Committee for Antimicrobial Susceptibility Testing (EUCAST).

All patients underwent arthroscopic surgery of the shoulder with drainage and debridement 24 h after diagnosis as an emergency treatment. Arthroscopy of the shoulder was performed in a beach-chair positiong. A standardized procedure was performed in every patient using the posterior, anterior and lateral portal [36]. A 70° arthroscopy was used for a better visualisation. Firstly, the subacromial space was examined before going through the rotator cuff into the glenohumeral joint.

Antibiotic treatment with cefuroxim 1.5 g intravenously began immediately after a diagnosis of septic arthritis as an emergency measure and ended when patients either deceased or gained normal CRP (normal reference value < 0.5 mg/dL), a normal leukocyte count (normal reference range 4.5–10 × 10^3^ cells/μL), and no fever for at least 48 h (<38.5 degrees Celsius, auricular). The antibiotic agent was changed if there was a positive microbiological culture indicating any resistance to cefuroxim. The antibiotic agent was administered for at least 7 days intravenously.

The probability of patient survival over a five-year period was estimated using Kaplan–Meier survival curves, separately for each of the following groups: older adults with septic arthritis with and without medical implants, with positive and negative synovial microbiological cultures, with or without osteoarthritis and grouped according to their preoperative ASA-PS classification scores (ASA II, ASA III, or ASA IV). These survival probabilities were compared by using the log-rank test.

The follow-up was determined from subsequent in-clinic medical records of following hospitalizations, public obituaries from newspapers or internet portals.

The study was approved by a university research ethics committee (IRB-2021-031, 9 November 2021). All data were collected and analyzed anonymously.

Microsoft Excel^TM^ 2016 and IBM SPSS Statistics version 28 were used for statistical analysis. The *p* value was considered significant at values of *p* ≤ 0.05.

Univariate analysis was performed and is stated as mean, median, standard deviation, and confidence interval unless otherwise noted.

## 3. Results

A total of 56 patients (37 males and 19 females) with a mean age of 78.7 years (range: 60–96 years) was included in the study. The mean follow-up time was 3011.8 days (8.25 years). The mean survival time of the entire study population was 920.3 days (2.52 years). The 1-year survival rate was 66.1%, and the 5-year survival rate was 34.4%.

Increasing age and higher ASA PS classification scores were significantly associated with impaired 5-year survival (*p* < 0.05). No significant differences in survival were found between patients with different types of surgical debridement and antibiotic regimens.

Eight different types of bacteria were detected in the synovial fluid cultures of 48 patients. *Staphylococcus aureus* was the most common pathogen, found in 20 patients (41.7%). Other Gram-positive bacteria included *Streptococcus* species (10 patients, 20.8%), *Enterococcus* species (6 patients, 12.5%), and coagulase-negative staphylococci (6 patients, 12.5%). Gram-negative bacteria, such as *Escherichia coli*, were less common (6 patients, 12.5%). No bacteria were identified in 8 patients (14.3%).

Detailed patient characteristics are demonstrated in Table 1.

### 3.1. Survival

The mean survival of the entire study population was 920.3 days, 2.52 years, (SD ± 158.4 days; 95% CI: 609.8–1230.7) and a median of 427 days with a SD ± 126.6 days.

**Figure 1:**
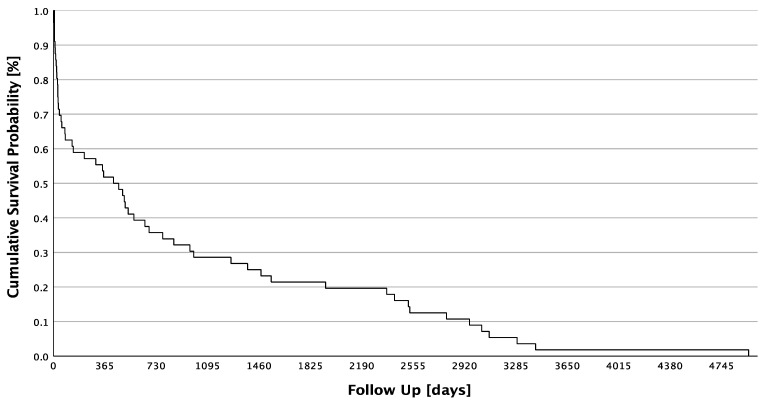
Cumulative Survival Probability of all patients.

The mean follow-up was 3011.8 days or 8.25 years (SD ± 2040.1 days; minimum: 323 days, maximum 7620 days). The Kaplan–Meier Survival analysis is shown in Figure 1. Overall, the successful follow-up rate was 89.3%. A total of 12 (21.4%) patients achieved a 5-year survival after their diagnosis of septic arthritis in the shoulder joint, whereas 44 (78.6%) patients did not survive 5 years. A total of 29 (21.4%) patients did not survive the first year after their diagnosis, and 27 (51.8%) patients were alive 1 year after diagnosis.

### 3.2. Microbial Spectrum

Eight different pathogens were detected in the analyzed probes. Of the eight organisms identified, five (62.5%) were Gram-positive and three (37.5%) were Gram-negative bacteria. Altogther, 42 (75%) of the patients were detected with Gram-positive bacteria, six (10.7%) with Gram-negative bacteria, and eight (14.3%) void of any microbial detection. More detailed information can be seen in Table 2.

**Figure 2:**
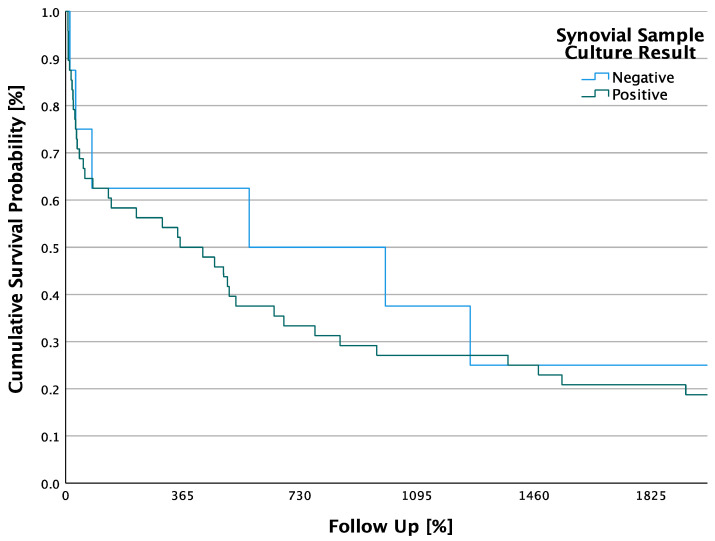
Cumulative 5-year survival probabilities of patients categorized as positive and negative synovial ample culture.

No significant difference in 5-year survival was observed between a positive and a negative synovial culture detection. The log-rank test calculated a *p*-value of 0.911 for 5-year survival between patients with a positive and a negative synovial detection, making the result statistically not significant. There was a total of eight (14.3%) patients without a pathogen detection in the synovial cultures. This group had a mean survival of 1034.8 days, 2.84 years, (SD ± 393.9 days; 95% CI: 262.7–1806.8). In 48 (85.7%) patients with bacterial septic arthritis, the median follow-up was 901.2 days, 2.47 years, (SD ± 174.2 days; 95% CI: 559.8–1242.6) (Figure 2). The 5-year survival rate without pathogen detection in synovial culture was 25% and with positive pathogen detection in synovial culture was 20.8%. 

### 3.3. ASA PS Classification

**Figure 3:**
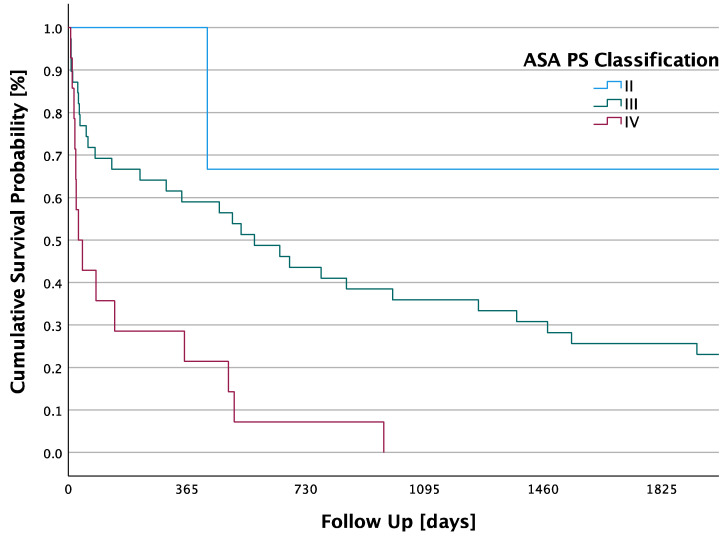
Cumulative 5-year survival probabilities of patients categorized as ASA PS classification II–IV.

Patients with an ASA II classification had a mean survival of 2631.7 days, 7.21 years, (SD ± 1302.9 days; 95% CI: 78–5185.3). Patients classified as ASA PS III had a mean survival of 1048.9 days, 2.87 years, (SD ± 182.2 days; 95% CI: 691.7–1406.1). Furthermore, the mean survival for patients in the ASA PS IV group was 195.2 days, 0.53 years, (SD ± 76.6 days; 95% CI: 45–345.4). A significant difference in 5-year survival was found between the different ASA PS classifications in ascending order with a *p*-value < 0.001. The 5-year survival rate in the ASA PS II group was 66.7%, in the ASA PS III group it was 25.6%, and in the ASA PS IV group it was 0%. Kaplan–Meier survival analysis is shown in Figure 3.

### 3.4. Number of Operations

No significant difference in 5-year survival was found between the numbers of operations. The log-rank test calculated a *p*-value of 0.561 for the different number of surgeries, making the result statistically not significant. There was a total of 26 (46.4%) patients with surgery. These had a median survival of 1004.4 days, 2.75 years, (SD ± 257.3 days; 95% CI: 500.1–1508.7). Some 20 (35.7%) patients had two operations for therapy for their septic arthritis. In this group, the median follow-up was 846.4 days, 2.32 years, (SD ± 227.2 days; 95% CI: 401.2–1291.6). Furthermore, 9 (16.1%) patients had three operations. There, the median follow-up time was 940.3 days, 2.58 years, (SD ± 433.7 days; 95% CI: 90.3–1790.3). In 1 (1.8%) patient with four operations, the follow-up was 31 days, 0.09 years, (SD ± 0 days).

**Figure 4:**
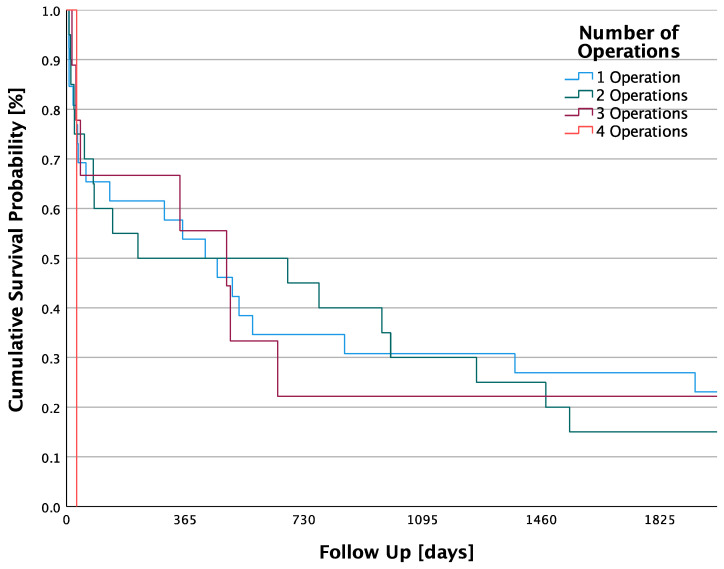
Cumulative 5-year survival probabilities of patients categorized as number of operations.

The 5-year survival rate in the one-surgery group was 26.9%, in the two-surgery group it was 15%, in the three-surgery group it was 22.2% and in the four-surgery group it was 0% (Figure 4).

## 4. Discussion

The main finding of this study is a very low survival rate of elderly patients receiving a diagnosis of primary septic arthritis of the shoulder. The most common causes are Gram-positive pathogens, with the most frequent being *Staphylococcus aureus*. Survival of elderly patients was limited with increasing ASA PS classification and with rising age.

Due to the scarce data about mid-to long term survival on septic arthitis of the shoulder, it is hard to compare survival data. The 1-year mortality was used, as this is most often used in studies of survival data. In this study, 1-year mortality was 48.2%. In patients with pre-existing osteoarthrosis of the shoulder, the 1-year mortality after a bacterial septic arthritis was 43.3%. Comparable studies of septic arthritis of the knee report values of 23.3% [37]. A national cohort study from Taiwan from 2020 stated that the overall 1-year survival for patients with native septic arthritis of the shoulder was 80.1%. Patients from the age of 18 years or older were included in this study. The different age structure makes a comparison difficult. This study also states that a shoulder infection and an age over 65 years is a negative predictive factor for patients with native septic arthritis [10].

The reason for differences in mortality rates could be based on the anatomic characteristics of the shoulder. The shoulder joint is separated into the glenohumeral joint and the subacromial space, which are separated by the rotator cuff. Infection can spread from one compartment to another. This favours a spreading of the infection even after operation and therefore it is more difficult to eradicate the infection. The clinical signs for a septic arthritis of the shoulder are just seen at a progredient state. This could be another reason for a higher mortality rate. Furthermore, the distribution of ASA classification between this study and the study of the septic arthritis of the knee varies significantly.

*Staphylococcus aureus* is the primary causative and most important pathogen. *Staphylococcus aureus* but additionally other bacteria such as *Staphylococcus epidermidis* or *Pseudomonas aeruginosa* form biofilms on implants. These biofilms are responsible for the persistence of implant infections and are a source of secondary systemic dissemination of bacteria [28,38]. This hematogenous dissemination of bacteria from biofilms is a potential cause of infection in septic shoulder arthritis. Furthermore, patients who have a medical implant have other preexisting conditions, making them more susceptible to infection and, theoretically, may have higher mortality.

This study revealed a significant difference in 5-year survival between the different ASA PS classifications. Thus, patients with an ASA PS II classification survived significantly longer than those with an ASA PS IV classification, who had the shortest survival. Several studies concluded that patients with higher ASA PS classifications had more frequent medical complications or mortality after surgery than those with lower ASA PS classifications [39,40,41,42]. Therefore, ASA PS classification has been reported to be a valuable prognostic variable for both postoperative medical complications and mortality [41,43]. Because patients classified as ASA PS IV suffer from severe systemic diseases and are operated only in emergencies, such as septic arthritis, operative and postoperative risks in these patients are very high. The increased mortality rate with a higher ASA PS classification was expected because these patients suffer from diseases which, even without septic arthritis, pose a permanent threat to life.

It is also remarkable that 26 (46.4%) patients received only one surgical treatment. In the literature, a number of required operations from one to four is recommended. It is suggested that the surgical treatment be repeated as often as required until the CRP value and the fever normalize [44]. Since this study retrospectively covered a period of more than 20 years, it must be taken into consideration that guidelines and literature recommendations differed over that time.

## 5. Limitations

The predicated survival rate for primary septic arthritis of the shoulder in this study is limited because of a missing control group with morbidity-related matched patients. In this way, the high mortality could also be based on the morbidity these patients had before suffering from primary septic arthritis. In future, there need to be studies investigating survival rates implementing a matched control group to announce the decline in survival rates in patients.

Limitations in terms of broader applicability, scientific accuracy, and interpretation of results, such as *p*-values and confidence intervals, arise from the single-center and retrospective study method [45]. Despite these limitations, this study design was chosen because primary septic shoulder arthritis, being a medical emergency, is a rare disease.

There is a a greater risk of the accumulation of errors because the data are not actively collected, but only taken from documents, such as medical records or findings. Thus, the collection of the data set, even if performed with the greatest possible care, is dependent on the available documents and data, and their quality and completeness. Moreover, each patient record was documented by different individuals and the time difference between individual patient records may be significant, resulting in important differences even within records. In addition, chart selection bias may occur because the data were collected and recorded without the intention of a study [46].

Actual survival or death were the only outcome included in the data. Underlying diseases, medications, and functional outcomes (with the exception of diabetes mellitus and osteoarthritis) were not included in this study. Individual direct causes of death also remain unknown as there were no further investigations after death, severely limiting the follow-up. Additionally, confounding factors are likely to exist in a geriatric population with various comorbidities that may not have been studied in detail.

In addition, this study considers a period of more than 20 years. During this time, diagnostic procedures and therapies changed. Furthermore, a standardized procedure for the detection and treatment of septic arthritis was introduced at a later time. Thus, there may be differences in the selection and accuracy of diagnostic procedures, and there may be a performance bias in therapy, as different physicians with different approaches and different levels of experience performed the operations.

The strength of this study is in the fact that the risk factors affecting mid-term survival of patients with septic arthritis were observed in conjunction with each other rather than individually and that follow-up was possible for a mid-term period in the majority of patients.

Age is a significant risk factor in osteoarthritis. This study did not investigate the presence of osteoarthritis in other joints [14,47]. Patients diagnosed with other types of osteoarthritis such as gout or pseudogout were excluded from the study. However, the presence of crystals in the synovial fluid does not exclude possible infection. Therefore, we excluded potentially eligible patients who were misdiagnosed with a primary diagnosis of crystal arthropathy and secondary septic arthritis.

Another limiting factor that must be considered is selection bias, as our follow-up rate of 89.3% may introduce survival bias because we do not have information on survival or death of those patients who were not followed up.

## 6. Conclusions

The median survival indicates that septic shoulder arthritis remains a condition with a high mortality, even if treated in a timely manner and adequately. Significantly decreased mid- and long-term survival was found with both increasing age and increasing ASA PS classification at the time of initial surgery. The ASA-PS classification can further help to predict the outcome and therefore to select the most appropriate therapy in order to enhance the chances of survival. The main causative agent for primary septic arthritis is the Gram-positive *Staphylococcus aureus*.

## Figures and Tables

**Table 1:** *n* = Number of patients; % = percentage; ASA PS Classification = American Society of Anesthesiologists Physical Status classification.

		Gender		Total
		*Male*	*Female*	
**Number of patients [*n*. (%)]**		37 (66.1)	19 (33.9)	56 (100)
**Age in years [Mean. Range]**		77 (61–95)	82 (64–100)	78.7 (61–100)
**Affected joint**	Left [*n* (%)]	13 (54.2)	11 (45.8)	24 (42.9)
	Right [*n* (%)]	24 (75)	8 (25)	32 (57.1)
**ASA PS Classification**	ASA I [*n* (%)]	0 (0)	0 (0)	0 (0)
	ASA II [*n* (%)]	2 (66.7)	1 (33.3)	3 (5.4)
	ASA III [*n* (%)]	22 (56.4)	17 (43.6)	39 (69.6)
	ASA IV [*n* (%)]	13 (92.9)	1 (7.1)	14 (25)
**Number of Operations [*n* (%)]**	1 [*n* (%)]	15 (57.7)	11 (42.3)	26 (46.4)
	2 [*n* (%)]	13 (65)	7 (35)	20 (35.7)
	3 [*n* (%)]	8 (88.9)	1 (11.1)	9 (16.1)
	4 [*n* (%)]	1 (100)	0 (0)	1 (1.8)
**Positive synovial fluid culture [*n* (%)]**		32 (66.7)	16 (33.3)	48 (85.7)
**Implants [*n* (%)]**		10 (55.6)	8 (44.4)	18 (32.1)
**Osteoarthritis of the shoulder [*n* (%)]**		16 (53.3)	14 (46.7)	30 (53.6)
**Diabetes mellitus [*n* (%)]**		10 (58.8)	7 (41.2)	17 (30.4)

**Table 2:** Distribution of bacteria in septic arthritis detected in synovial fluid cultures. *MRSA* = *Methicillin-resistant Staphylococcus aureus*.

Pathogen	*n* (%)
**Gram-positive**	
*Staphylococcus aureus*	31 (55.4)
MRSA	3 (5.4)
*Staphylococcus epidermidis*	3 (5.4)
*Propionibacterium acnes*	3 (5.4)
*Enterococcus faecalis*	2 (3.6)
Total Gram-positive	42 (75)
**Gram-negative**	
*Escherichia coli*	4 (7.1)
*Pseudomonas aeruginosa*	1 (1.8)
*Citrobacter freundii*	1 (1.8)
Total Gram-negative	6 (10.7)
**Negative synovial fluid culture, 8 (14.3)**	
**Total**	56 (100)

## Data Availability

The data originate from a surgical and traumatological maximum care unit at Klinikum Nürnberg. The data can be provided anonymously in a separate file upon request. A public dataset was not used to obtain the data presented.
